# Ubiquitin-specific protease 28: the decipherment of its dual roles in cancer development

**DOI:** 10.1186/s40164-023-00389-z

**Published:** 2023-03-06

**Authors:** Xiaoya Ren, Menglong Jiang, Peng Ding, Xiaoyan Zhang, Xin Zhou, Jian Shen, Dong Liu, Xiaolong Yan, Zhiqiang Ma

**Affiliations:** 1 Department of Thoracic Surgery, Tangdu Hospital, Air Force Medical University, 1 Xinsi Road, Xi’an, 710038 China; 2 Department of Medical Oncology, Senior Department of Oncology, Chinese PLA General Hospital, The Fifth Medical Center, 28 Fuxing Road, Beijing, 100853 China; 3 Department of Thoracic Surgery, 1st Affiliated Hospital of Anhui Medical University, Hefei City, China; 4 Department of Aerospace Medicine, Air Force Medical University, Xi’an, China; 5 Senior Department of Cardiology, The Sixth Medical Center, Chinese PLA General Hospital and Chinese PLA Medical School, 28 Fuxing Road, Beijing, 100853 China; 6 State Key Laboratory of Cardiovascular Disease, Fuwai Hospital, National Center for Cardiovascular Diseases, Chinese Academy of Medical Sciences, Peking Union Medical College, 167 Beilishi Road, Beijing, 100037 China

**Keywords:** Biomarker, USP28, Cancer, Prognosis, Therapy resistance

## Abstract

As significant posttranslational modifications, ubiquitination and deubiquitination, whose balance is modulated by ubiquitin-conjugating enzymes and deubiquitinating enzymes (DUBs), can regulate many biological processes, such as controlling cell cycle progression, signal transduction and transcriptional regulation. Belonging to DUBs, ubiquitin-specific protease 28 (USP28) plays an essential role in turning over ubiquitination and then contributing to the stabilization of quantities of substrates, including several cancer-related proteins. In previous studies, USP28 has been demonstrated to participate in the progression of various cancers. Nevertheless, several reports have recently shown that in addition to promoting cancers, USP28 can also play an oncostatic role in some cancers. In this review, we summarize the correlation between USP28 and tumor behaviors. We initially give a brief introduction of the structure and related biological functions of USP28, and we then introduce some concrete substrates of USP28 and the underlying molecular mechanisms. In addition, the regulation of the actions and expression of USP28 is also discussed. Moreover, we concentrate on the impacts of USP28 on diverse hallmarks of cancer and discuss whether USP28 can accelerate or inhibit tumor progression. Furthermore, clinical relevance, including impacting clinical prognosis, influencing therapy resistance and being the therapy target in some cancers, is depicted systematically. Thus, assistance may be given to future experimental designs by the information provided here, and the potential of targeting USP28 for cancer therapy is emphasized.

## Background

Ubiquitin-specific protease 28 (USP28), a significant deubiquitinating enzyme, plays an essential role in antagonizing the effects of E3 ligases. It has been demonstrated to play an indispensable role in stabilizing oncoproteins and thus participating in the advancement of cancers. Nevertheless, several reports have recently illustrated the oncostatic actions of USP28 in some cancers. In this review, we summarize the correlation between USP28 and tumor behaviors, aiming to provide guidance for future experimental designs and emphasize the potential of targeting USP28 for cancer therapy.

## Introduction

As a posttranslational modification (PTM), ubiquitination alters vital properties of substrate proteins, including their activity and half-life in cells, thus playing a significant role in many biological processes, such as controlling cell cycle progression, signal transduction and transcriptional regulation [1]. Similar to phosphorylation, which is also a major PTM, protein ubiquitination can be controlled by counterbalancing the action of various ubiquitin conjugating enzymes and deubiquitinating enzymes (DUBs) [2]. As a DUB, USP28, which was discovered due to its homology with USP25, can contribute to protein deubiquitination as well [3]. Scientists have demonstrated that USP28 can reverse the ubiquitination of many substrates and then participate in related cellular processes, including apoptosis [4], cell proliferation [5], stress response [6] and DNA damage response [7]. Since its involvement in many significant processes, dysregulation of USP28 has been illustrated to be associated with various cancers [4, 8–11].

During the past few decades, the role of USP28 in promoting cancers has attracted scientists’ attention [11]. In addition to the higher expression of USP28 discovered in a variety of cancers, including glioma and non-small cell lung cancer, a negative correlation between the level of USP28 and the prognosis of these cancers has been demonstrated [12, 13]. Moreover, some molecules, such as streptoglutarimide H and AZ1, which can contribute to the decrease in USP28, have the capacity to inhibit the progression of cancers [14, 15]. That is, dysregulation of USP28 has been proven to accelerate the progression of many cancers, and targeting it for cancer therapy may be a potential direction. However, apart from promoting the progression of cancers, some recent studies have revealed that USP28 can participate in the suppression of some cancers involving melanoma [16]. Therefore, further investigation regarding USP28 and its impacts on cancers is still needed.

In this review, we systematically introduce recent investigations regarding the relationship between USP28 and cancer. Initially, a brief introduction of the structure and biological functions of USP28 is given, based on which we then introduce some substrates of USP28. Subsequently, we depict pathways that can regulate the expression and actions of USP28. Then, we especially emphasize the role USP28 plays in the dual regulation of cancer hallmarks. The influence of USP28 on cancer prognosis and drugs that can target USP28 in carcinomas is concluded next. Ultimately, numerous prospective research fields related to USP28 and cancers are offered with detailed analysis. Collectively, serving as a reference for investigators in relevant fields of study, the information compiled here may aid in future investigations, especially in whether USP28 can be a target for the treatment of diverse cancers.

### Molecular features and biological functions of USP28

**Fig. 1:**
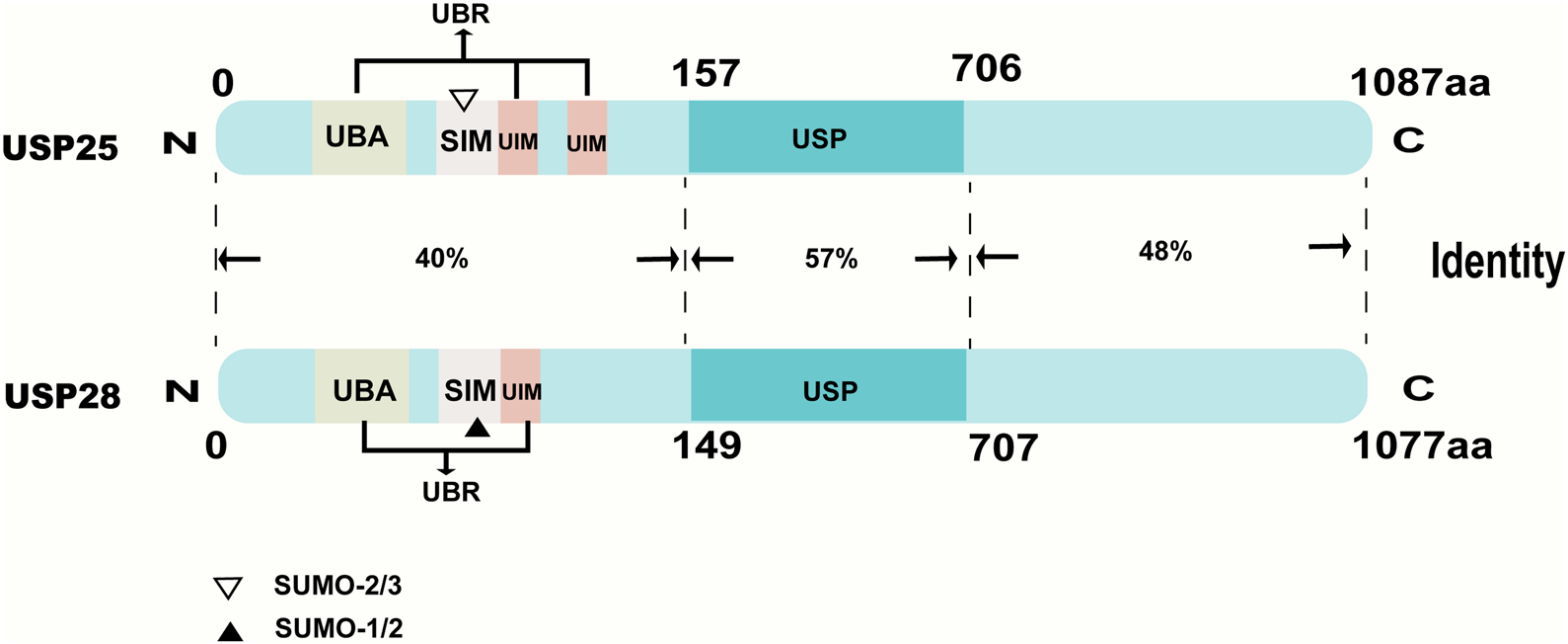
Structures of USP28 and USP25. Both of USP28 and USP25 possess the N-terminal domain, the catalytic “conserved” USP domain and the C-terminal extension domain and the identity of the three domains between USP28 and USP25 is 40%, 57% and 48% respectively. Except for the shared domains including one UBA, one SIM and one UIM, the N-terminal of USP25 has another more UIM compared to USP28. Domains including UBA and UIM compose of UBR, which is responsible for ubiquitin recognition. As for SIM, USP28's SIM prefers to interact with SUMO-1/2 while USP25's prefers to interact with SUMO-2/3. *SIM* SUMO-interaction motif, *UBA* ubiquitin-associated domain, *UBR* ubiquitin-binding region, *UIM* ubiquitin-interaction motif, *USP* ubiquitin-specific protease

USP28, mapped to chromosome 11q23, was discovered because of its homologous sequence with USP25. With a length of at least 3624 bp, USP28 cDNA encodes 1077 amino acids with a relative molecular mass of 122.4 kDa [3]. The whole protein can be divided into the N-terminal domain, the catalytic “conserved” USP domain and the C-terminal extension domain [17]. Composed of a ubiquitin-binding region (one ubiquitin-associated domain [UBA] and one ubiquitin-interaction motif [UIM]) and one SUMO2/3-selective SUMO-interaction motif (SIM), the N-terminal domain of USP28 shares 40% identity with USP25 [18, 19] (Fig. 1). The catalytic domain of USP28 incorporates four subdomains, including the thumb, palm, finger and USP28 catalytic domain inserted domain (UCID). The subdomains, except for UCID, form the catalytic USP domain, which can bind to ubiquitin. UCID is a domain that consists of a lower “rod” (Lys402-Ser453; Asp535-Gln572) and an upper “tip” (Thr454-Thr534), and the upper part of the “rod” is involved in the achievement of USP28 dimerization. Moreover, resulting from the significant differences in close proximity to the active sites between USP28 and USP25, USP28 is sixfold more active and efficient than USP25 in catalytic activities [19].

**Fig. 2:**
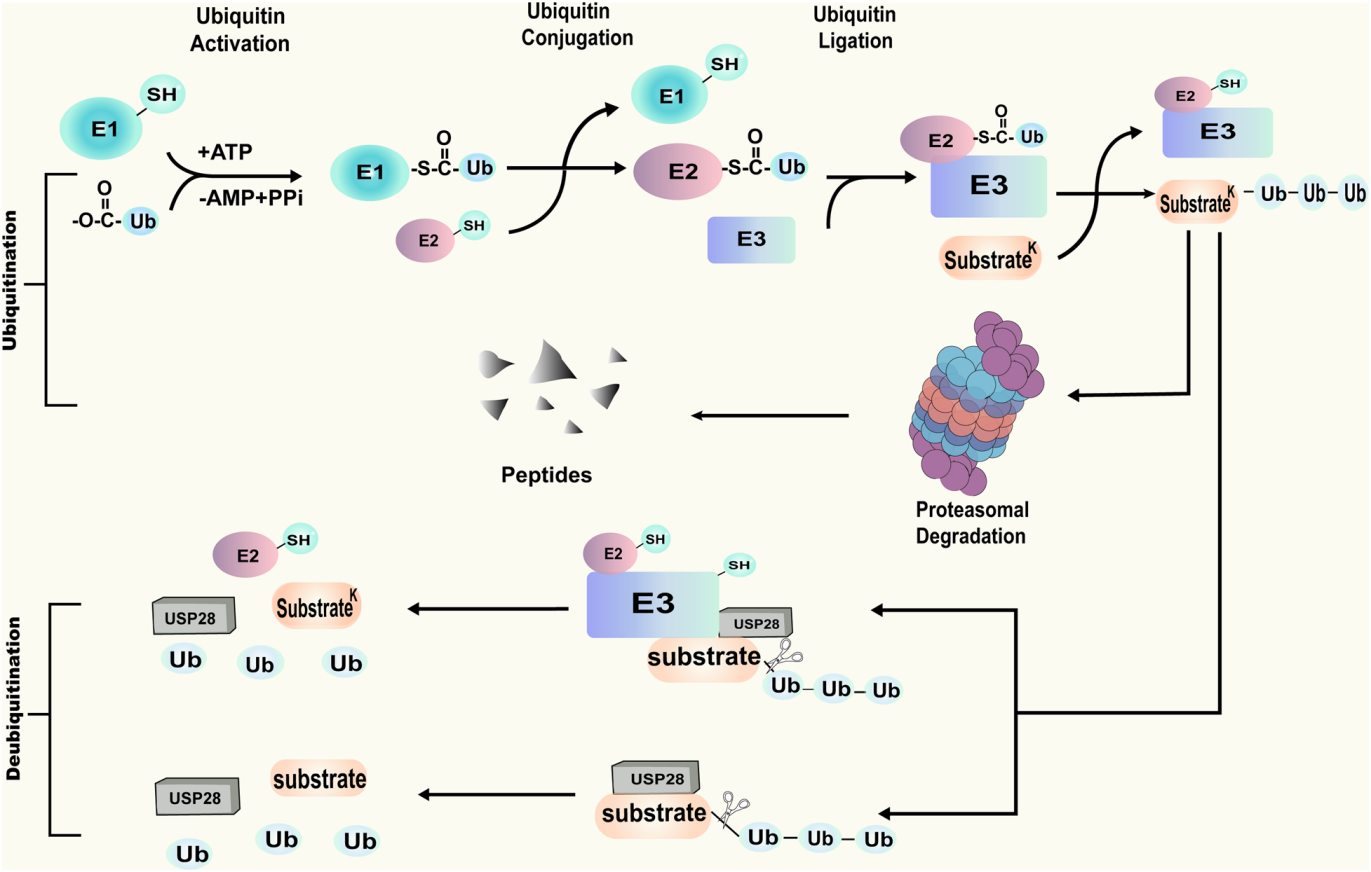
Schematic introduction of ubiquitination and deubiquitination. Sequential catalyzation caused by E1, E2 and E3 contributes to ubiquitin activation, ubiquitin conjugation and ubiquitin ligation respectively, leading to the ubiquitination of substrates. Ubiquitination of substrates results in the proteasomal degradation of themselves. While if deubiquitinases such as USP28 exist, the ubiquitin chains of substrates can be removed and the interaction between the enzyme and the substrates is realized directly or indirectly (with the help of E3 ligases)

After the description of the structure of USP28, its biological functions are ready for introduction. As one of the posttranslational modifications, ubiquitination involves covalent conjugation of ubiquitin to lysine (K) residues of target proteins, a process that is catalyzed by the sequential action of ubiquitin-activating enzymes (E1 s), ubiquitin-conjugating enzymes (E2 s) and ubiquitin-ligating enzymes (E3 s) [1, 20]. DUBs that can antagonize ubiquitination are classified into 7 subfamilies based on distinct structural and functional features, among which USPs represent the bulk [21–24]. Moreover, database homology searches with the reported USP25 contribute to the discovery of USP28, and it is a new USP member with demonstrated ubiquitin-specific protease activity [3] (Fig. 2).

### Substrates of USP28

Studies have shown that USP28 possesses the ability to turn over the ubiquitination caused by many E3 ligases, such as F-box and WD repeat domain-containing protein 7 (FBW7) [25], mouse double minute 2 protein (MDM2) [26], and Kelch-like protein 2 (KLHL2) [27]. Among all the E3 ligases discussed, FBW7 is one of the most significant, and not only its substrates but also itself can be deubiquitinated by USP28. Moreover, a lower level of USP28 is required for deubiquitination of FBW7 than FBW7 substrates [28].

Among the molecules that are ubiquitinated by FBW7, c-Myc is also one of the substrates of USP28 [29]. A study showed that the depletion of FBW7 reduced the connection between USP28 and c-Myc, indicating that FBW7 mediated the interaction between them [5]. Past investigations revealed that although many FBW7 domains, including WD40, promoted FBW7’s binding to USP28, the amino terminus of FBW7 contributed to the interaction of FBW7, c-Myc and USP28 [5, 29]. Regarding the connection between FBW7 and c-Myc, phosphorylation at c-Myc Thr58 gives rise to c-Myc’s connection with FBW7 [30]. However, in the absence of FBW7, the direct interaction of USP28 and c-Myc was also observed, during which USP28 was more likely to bind to unphosphorylated peptide [31]. For example, under epinephrine treatment, USP28 Cys^171^ has the capacity to interact directly with the c-Myc MBI domain [6]. Both the direct and indirect interactions between USP28 and c-Myc contribute to the deubiquitination and stabilization of c-Myc. Similar to c-Myc, hypoxia-inducible factor-1α (HIF-1α) is also one of the substrates of USP28 and FBW7, and the deubiquitination of HIF-1α catalyzed by USP28 requires the mediation of FBW7 as well. Moreover, the interaction between FBW7 and HIF-1α requires the phosphorylation of HIF-1α by glycogen synthase kinase-3β (GSK-3β) [25].

In addition to turning over the ubiquitination caused by FBW7, USP28 can also reverse the function of MDM2. Both p53 and ΔNp63 can be deubiquitinated by USP28 by removing their K48-linked ubiquitin chains, which are induced by MDM2 [15, 26]. Additionally, the level and activity of the histone demethylase lysine specific demethylase 1 (LSD1) can be controlled by USP28 through a deubiquitination event, during which an amine oxidase domain in LSD1 and the N-terminal region of USP28 are responsible for the LSD1 interaction with USP28 [9]. Similar to removing the K-48 linked ubiquitin chains of p53, studies have illustrated that USP28 can interact with H2A directly and reduce the level of ubiquitination at K119 of histone H2A (ub-K119-H2A) [32]. Additionally, USP28 can reverse the polyubiquitination of uridine cytidine kinase 1 (UCK1) at K81 catalyzed by KLHL2, which is required for the link between USP28 and KLHL2 [27].

Apart from the molecules discussed above, USP28 has been proven to deubiquitinate many substrates, such as signal transducer and activator of transcription 3 (STAT3) [33], Cyclin E1 [34], NOTCH1 intracellular domain (NICD1) [35], ΔNp63 [15], cluster of differentiation-44 (CD44) [36], Claspin [37] (Table 1). Nevertheless, what is different from the molecules discussed above is that the detailed mechanisms and the specific site or region of the interaction between these substrates and USP28 have not been explored.

**Table 1:** Actions of USP28 on its potential substrates in cancers

Target of USP28	Function of USP28	The region and site involved	Results of the USP28 effects on the target	References
FBW7 (FBXW7)	Antagonizing its autocatalytic ubiquitination	–	Degradation of oncoproteins	[28]
c-Myc	Countering the ubiquitination mediated by FBW7	amino-terminus of FBW7; p–c-Myc-T58	Promoting the cell cycle and glycolysis	[5, 29, 30]
		MBI domain of MYC; USP28 Cys171	Transactivating the SLUG promoter to enhance cancer stem-like traits	[6, 31]
c-Jun	Inhibiting the functions of FBW7, deubiquitinating and stabilizing c-Jun	–	Accelerating cell proliferation	[35]
NICD1	Repressing functions of FBW7, deubiquitinating NICD1	–	Strengthening Notch signaling and influencing the secretory fate determination of intestine	[35]
HIF-1α	Revising the destabilization caused by FBW7, and promoting its accumulation through c-Myc	Closely related to the GSK-3β phosphorylation sites	Expediting the angiogenesis	[25]
Cyclin E1	Loss of USP28 that contributes to the autoubiquitination of FBW7 and overexpression of USP28 can stabilize it	–	Promoting cell cycle progression	[34]
STAT3	Upregulating STAT3 and reversing the polyubiquitination mediated by FBW7	–	Promoting cellular growth	[33]
p53	Deubiquitination p53 through antagonizing MDM2	MDM2-catalyzed K48 ubiquitin chains	Inducing apoptosis	[26]
ΔNp63	Removing the K-48 linked ubiquitin chains independently of FBW7	MDM2-catalyzed K48 ubiquitin chains	Facilitating cell proliferation and maintaining identity of squamous cancer cells	[25]
LSD1	Deubiquitinating and stabilizing it	An amine oxidase domain of LSD1; N-terminal region of USP28	Suppressing differentiation, promoting cell proliferation and metastasis	[9, 55]
CD44	Removing ubiquitin group from CD44 protein and enhancing its stabilization	–	Maintaining the stem cell-like properties and promoting invasion	[36]
H2A	Binding H2A and deubiquitinating it	ub-K119-H2A	Suppressing cell proliferation	[32]
Claspin	Deubiquitinating and stabilizing it	–	Maintaining cell cycle arrest and cell survival in response of DNA damage caused by chemotherapy	[37]
Snail	Stabilizing Snail	–	–	[82]
Lin28A	Extending the half-life of Lin28A and stabilizing it	–	Promoting cell viability, colony formation and invasion	[74]
ZNF304	Decreasing ubiquitination of it	–	Switching of many tumor suppressor genes	[47]
MDC1	Leading to its stabilization	–	Promoting rescue from damage or contributing to apoptosis	[7]
UCK1	Antagonizing its ubiquitination caused by KLHL2	K81 of UCK1	Giving rise to the chemotherapeutic resistance to 5’AZA	[27]
CHK2	Turning over its ubiquitination mediated by PIRH2	–	Enhancing the activation of G1/S and G2/M checkpoints	(141)
Plk3	Antagonizing the suppression function of SIAH2	–	Inhibiting carcinogenesis	[111]
FOXM1	Directly interacting with it and promoting its stabilization	–	Promoting cell proliferation and inhibiting apoptosis	[4]

*CD44* cluster of differentiation-44, *CHK2* checkpoint kinase 2, *FBW7* F-box and WD repeat domain-containing protein 7, *FOXM1* Forkhead box M1, *GSK-3* glycogen synthase kinase-3, *HIF-1α* hypoxia-inducible factor-1α, *KLHL2* Kelch-like 2, *LSD1* lysine specific demethylase 1, *MB I* MYC Box I, *MDC1* mediator of DNA damage checkpoint 1, *MDM2* mouse double minute 2 protein, *NICD1* NOTCH1 intracellular domain, *PIRH2* p53-induced RING-H2, *Plk3* polo-like kinase 3, *STAT3* signal transducer and activator of transcription 3, *ub-K119-H2A* ubiquitination at K119 of histone H2A, *UCK1* uridine-cytidine kinase, *USP28* ubiquitin-specific protease 28, *ZNF304* zinc finger protein 304

### Regulatory mechanisms of USP28 expression

Many studies have demonstrated that miRNAs can form miRNA-induced silencing complexes, inhibiting mRNA translation or accelerating RNA degeneration [38, 39]. In breast cancer cell lines, a reduction in USP28 was observed following miR-500a-5p mimic transfection because miR-500a-5p could target USP28 at two potential binding sites in the 3’-UTR of USP28. Moreover, MTT assays revealed that the functions of USP28 overexpression could be partially turned over by miR-500a-5p mimic transfection [40]. That is, USP28 is a direct target of miR-500a-5p. Apart from miR-500a-5p, miR-3940-5p, which has been demonstrated to be decreased in non-small cell lung cancer, has the capacity to connect with the 3’-UTR of USP28 and then lead to the degradation and repression of USP28 [41, 42]. Additionally, as one of the substrates of USP28, c-Myc can suppress miR-363-3p. However, ectopic expression of miR-363-3p in human hepatocellular carcinoma cell lines causes a decrease in USP28, indicating that USP28, c-Myc and miR-363-3p constitute a negative feedback loop [8].

**Fig. 3:**
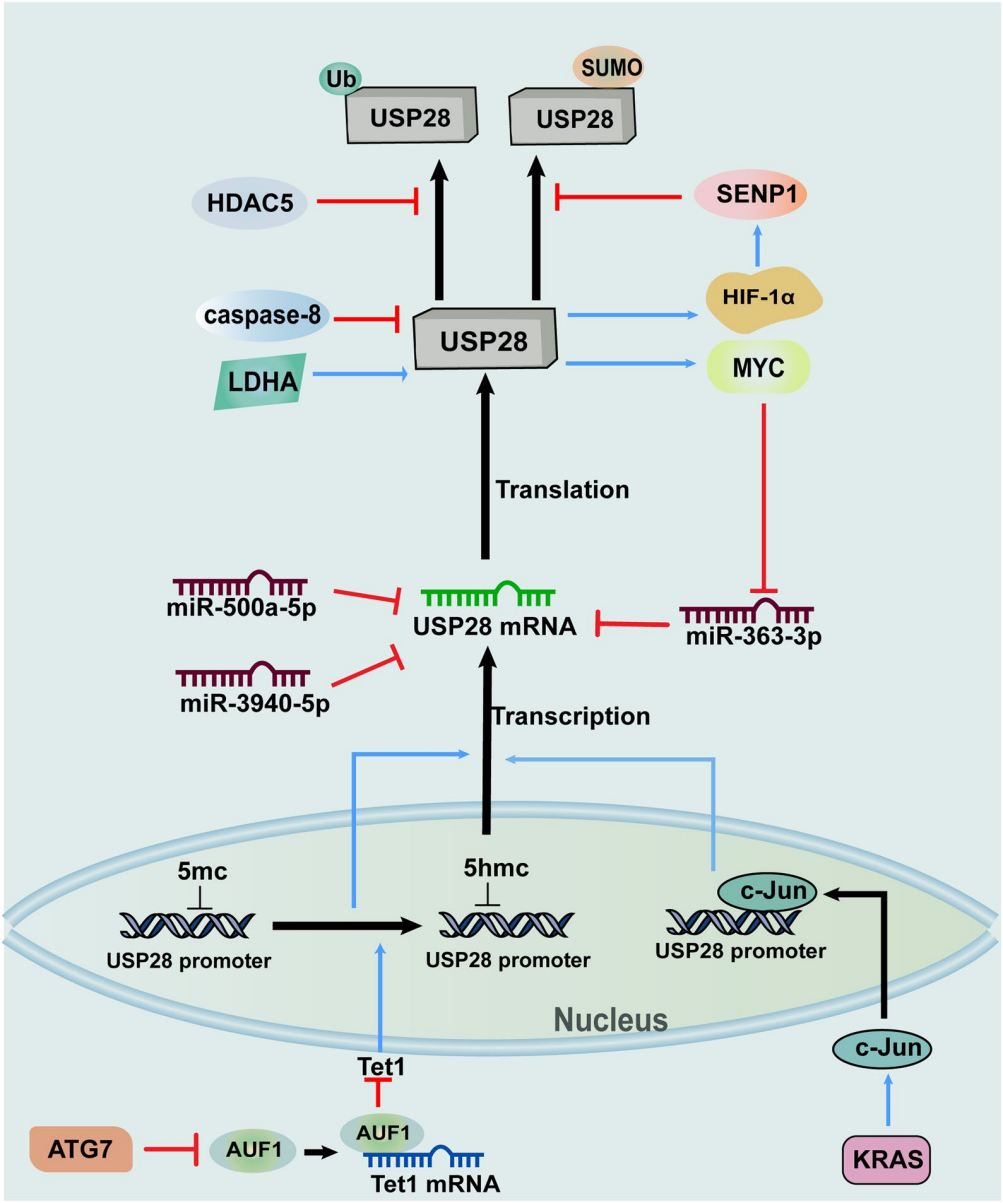
Regulatory mechanisms of the stability, activation and expression of USP28. During the expression of USP28, both Tet1 and c-Jun can function on USP28 promoter and contribute to the enhancement of USP28 transcription. While miRNAs involving miR-363-3p which can be inhibited by USP28 substrate MYC, miR-500a-5p and miR-3940-5p have the capacity to interact with USP28 mRNA and then inhibit the translation of USP28. SUMOylation and LDHA can respectively suppresses and promotes the activation of USP28, and the former one can be antagonized by SENP1. Moreover, USP28 substrate HIF-1α can induce SENP1, thus forming a feedback loop. Besides, ubiquitination and caspase-8 can induce the destabilization of USP28, and HDAC5 brings about the inhibition of USP28 ubiquitination. *ATG7* autophagy related protein 7, *AUF1* ARE/poly(U)-binding/degradation factor 1, *HDAC5* histone deacetylase 5, *HIF-1α* hypoxia-inducible factor-1α, *LDHA* lactate dehydrogenase A, *SENP1* SUMO-specific protease 1, *SUMO* small ubiquitin-like modifier, *Tet1* Ten-eleven translocation 1, *Ub* ubiquitin, *USP28* ubiquitin-specific protease 28

Ten-eleven translocation1 (Tet1), a DNA demethylase, can promote the expression of USP28 through facilitating demethylation of USP28 promoter. Besides, study also shown that autophagy related-protein 7 (ATG7) enhanced autophagy of ARE/poly(U)-binding/degradation factor 1 (AUF1) protein, which caused the increased level of Tet1 [43]. Another molecule, KRAS, which plays a vital role in the signal transduction of most growth factor receptors, has been validated to increase the activity of c-Jun and upregulate USP28 [44–47]. Furthermore, the reduction in USP28 in cells with c-Jun loss and the CHIP results indicate that c-Jun can make a connection with the USP28 promoter, revealing that c-Jun is responsible for the upregulation of USP28 caused by KRAS [47] (Fig. 3).

### Regulatory mechanisms of USP28’s actions

Protein conjugation with small ubiquitin-like modifier (SUMOylation), a posttranslational modification, regulates diverse proteins through many different pathways [48, 49]. A study determined that SUMOylation of USP28 usually occurs at lysine 99, which is in the UIM domain of the USP28 N-terminus. SUMO can suppress the activation of USP28 through direct interaction with the catalytic domain of USP28 [17, 50]. Additionally, similar to deubiquitination induced by USP28, SUMOylation of USP28 can be revised by SUMO-specific proteases (SENPs), and expression of SENP1 can be induced by the USP28 substrate HIF-1α [51, 52]. Thus, the positive feedback loop consisting of SENP1, USP28 and HIF-1α is reasonably demonstrated [53].

Moreover, the half-life and expression of USP28 were observed to be reduced when lactate dehydrogenase A (LDHA) was knocked down. Interestingly, LDHA can bring about a weakly acidic environment in which the signal transduction of USP28 is strengthened and the distance between USP28 and its substrates is shortened, indicating that LDHA has the capacity to enhance the deubiquitination function of USP28 [6]. Additionally, belonging to class IIa HDACs, histone deacetylase 5 (HDAC5) attenuates the polyubiquitination of USP28 through its direct interaction with USP28, suggesting that HDAC5 has the ability to stabilize USP28 [54–56]. In addition to the molecules discussed above, the cysteine-aspartate-specific protease caspase-8 can recognize USP28 and inactivate it through the cleavage sites _115_IQAD_120_ and _236_AALD_241_ in USP28 [26] (Fig. 3).

### Oncogenic functions of USP28 in tumors

**Fig. 4:**
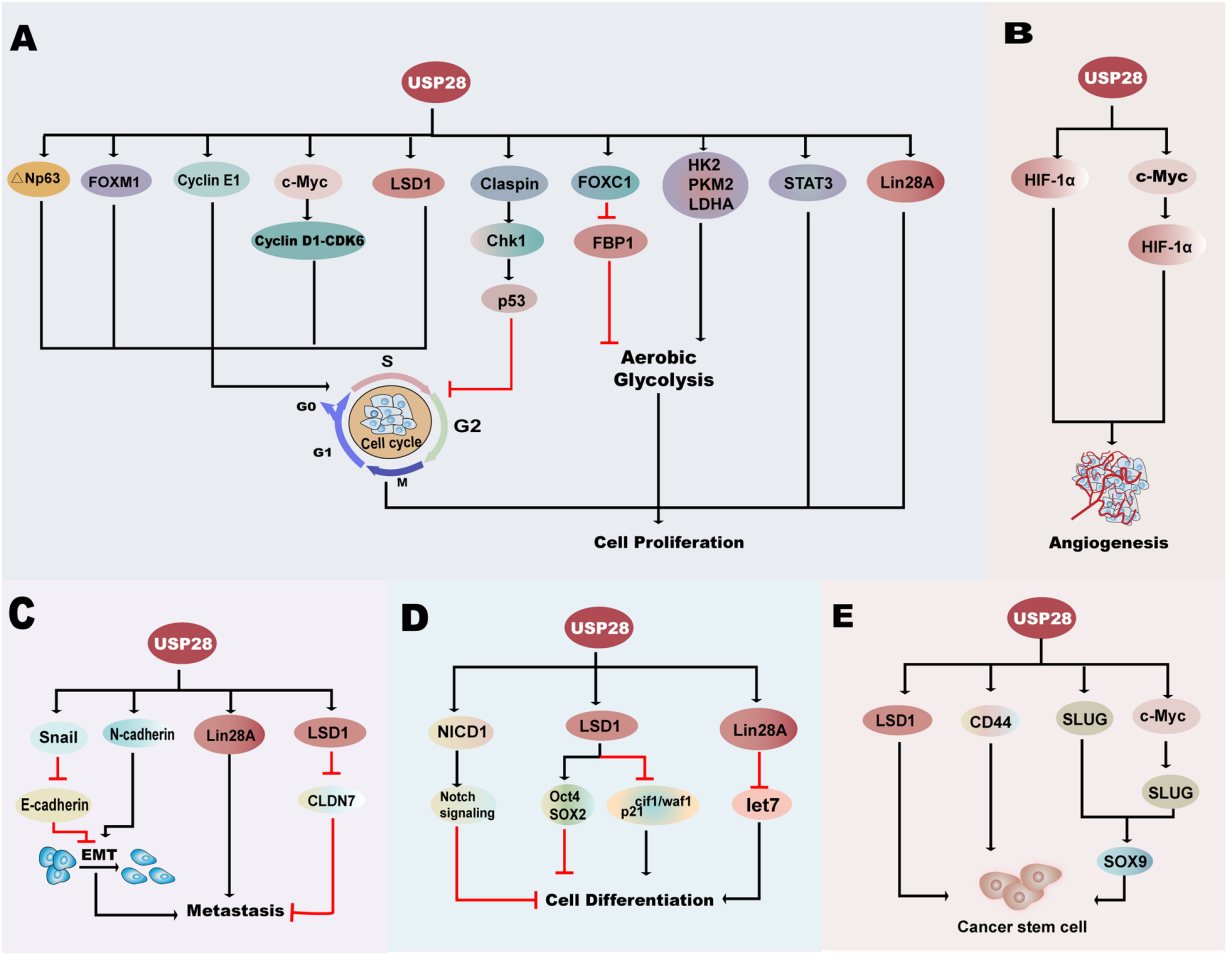
The underlying mechanisms of USP28 in promoting cancer progression. USP28 can promote the development of cancer through deubiquitinating lots of substrates related to tumor progression. **A** USP28 can promote the progression of cell cycle, aerobic glycolysis and then contribute to the enhancement of cell proliferation. **B** USP28 accelerates angiogenesis through the mediation of HIF-1α and c-Myc. **C** Promotion of EMT and metastasis can be induced by USP28. **D** USP28 suppresses cell differentiation through its deubiquitination on NICD1, LSD1 and Lin28A. **E** USP28 functions on maintaining cancer stem cell-like characteristics. *CD44* cluster of differentiation-44, *CDK6* Cyclin-dependent kinase 6, *Chk1* checkpoint kinase 1, *CLDN7* claudin-7, *CSC* cancer stem cell, *EMT* epithelial-mesenchymal transition, *FBP1* fructose 1,6-bisphosphatase 1, *FOX* the Forkhead box, *HIF-1α* hypoxia-inducible factor-1α, *HK2* hexokinase 2, *LDHA* lactate dehydrogenase A, *let-7* lethal-7, *LSD1* lysine specific demethylase 1, *NICD1* NOTCH1 intracellular domain, *Oct4* octamer-binding transcription factor 4, *PKM2* M2 isoform of pyruvate kinase, *SOX* SRY-related HMG box-containing, *STAT3* signal transducer and activator of transcription 3, *USP28* ubiquitin-specific protease 28

Many studies have demonstrated that USP28 can promote the deubiquitination and stabilization of many oncoproteins, implying that USP28 can stimulate the progression of many tumors. To further understand the effects of USP28 in diverse cancers and its potential significance in clinical applications, we concentrate on the roles USP28 plays in different hallmarks of cancers (Fig. 4; Table 2).

**Table 2:** Functions of USP28 in cancers

Effect of USP28	Effect of USP28 on cancer hallmarks	Cancer type	Related mechanisms	The effects of USP28 expression in cancer prognosis	References
Promoting cancers	Promoting cell proliferation	Ovarian clear cell carcinoma	HNF-1β upregulates USP28, which gives rise to the high level of Claspin and CHK1 after the treatment of bleomycin	–	[37]
		High grade serous ovarian cancer	USP28 increases the level of Cyclin E1, which then promotes cell cycle	–	[34]
		Squamous cancer	Elevated USP28 upregulates ΔNp63 and participates in the process of pro-cancer with mediation of ΔNp63	Negative	[15]
		Pancreatic cancer	USP28 stabilizes FOXM1 and then leads to the activation of Wnt/β-catenin pathway	Negative	[4]
		Lung cancer	USP28 deubiquitinates c-Myc, through which the activation of Cyclin D1-CDK6 complex is promoted. c-Myc is stabilized by USP28 and then increases HK2, PKM2 and LDHA, stimulating aerobic glycolysis	–	[14]
			USP28 enhances STAT3 signaling	Negative	[33]
		Breast cancer	Overexpression of HDAC5 reduces the polyubiquitination of USP28, contributing to the increase of LSD1	–	[55]
		Colorectal cancer	USP28 maintains the expression of FOXC1 and then leads to the decrease of FBP1, enhancing aerobic glycolysis	–	[71]
		–	Lin28A brings about more colonies with the help of USP28	–	[74]
	Initiating invasion and metastasis	Gastric cancer	USP28 contributes to higher N-cadherin while lower E-cadherin, leading to the stimulation of EMT	–	[83]
			USP28 stabilizes Snail proteins which may then suppresses E-cadherin	–	[80, 82]
		–	Deubiquitination of Lin28A caused by USP28 promotes metastasis	–	[74]
		Breast cancer	Stabilization of LSD1 mediates the process that USP28 inhibits CLDN7	–	[55]
	Inhibiting cell differentiation	Colorectal cancer	USP28 stabilizes NICD1 and maintain the activation of Notch signaling, leading to the suppression of cell differenciation	Negative	[35]
		Breast cancer	USP28 deubiquitinates LSD1, and then differentiation gene p21^Cif1/Waf1^ reduces while pluripotency markers SOX2 and Oct4 increases	–	[9]
		–	USP28 enhances the suppression of let-7 induced by Lin28A, contributing to the suppression of cell differentiation	–	[74]
	Inducing angiogenesis	–	USP28 antagonizes the impact of FBW7 and inhibits the ubiquitination of HIF-1α	–	[25]
		Esophageal cancer	c-Myc can be deubiquitinated by USP28 and then results in the accumulation of HIF-1α	–	[97]
	Maintaining cancer stem cell-like characteristics	Bladder cancer	Through upregulation USP28, ATG7 promotes the expression of CD44	-	[36]
		Breast cancer	USP28 contributes to the stabilization of c-myc, which stimulates the upregulation of SLUG and then maintains CSCs mediated by SOX9	-	[6, 101]
			USP28-LSD1 axis protects characteristics of CSCs	–	[9]
Inhibiting cancers	Inhibiting cell proliferation	–	Knockdown of caspase-8 facilitates activation of USP28 which then brings about p53 stabilization	–	[26]
		–	USP28 decreases ub-K119-H2A, leading to the elevation of p53, p21 and p16^INK4A^	–	[32]
		–	USP28 turns over the ubiquitination of Plk3 possessing the ability to activate CHK2 and p53	–	[111]
		Melanoma	USP28 reverses the auto-ubiquitination of FBW7, resulting in the decrease of BRAF and weakness of MAPK signaling	Positive	[16]
	Suppressing tumor invasion and metastasis	Breast cancer	USP28 reduces the accumulation of α-SMA while increases E-cadherin	Positive	[10]
			Overexpression of USP28 leads to decrease of FN-1 and elevation of E-cadherin	–	[40]
		Melanoma	USP28 reverses the auto-ubiquitination of FBW7, resulting in the decrease of BRAF and weakness of MAPK signaling	Positive	[16]

*ATG* autophagy related-protein, *BRAF* v-raf murine sarcoma viral oncogene homolog B, *CD44* cluster of differentiation-44, *CDK6* Cyclin-dependent kinase-6, *Chk1* checkpoint kinase 1, *CLDN7* tight junction protein claudin-7, *CSC* cancer stem cell, *EMT* Epithelial–mesenchymal transition, *FBP1* fructose-1,6-bisphosphatase 1, *FBW7* F-box and WD repeat domain-containing protein 7, *FOX* Forkhead box, *HDAC* histone deacetylases, *HIF-1α* hypoxia-inducible factor-1α, *HK2* hexokinase 2, *LDHA* lactate dehydrogenase A, *let-7* lethal-7, *LSD1* lysine specific demethylase 1, *MAPK* mitogen-activated protein kinase, *NICD1* NOTCH1 intracellular domain, *Oct4* octamer-binding transcription factor 4, *PKM2* M2 isoform of pyruvate kinase, *Plk3* polo-like kinase 3, *α-SMA* α-smooth muscle actin, *SOX* SRY-related HMG box-containing, *STAT3* signal transducer and activator of transcription 3, *ub-K119-H2A* ubiquitination at K119 of histone H2A, *USP* ubiquitin-specific protease

#### Promoting cell proliferation in tumorigenesis

In normal tissues, growth-promoting signals that stimulate the cell progress-and-division cycle are controlled to guarantee the homeostasis of the number and the functions of the cells. Nevertheless, deregulation of these signals occurs in cancer cells and participates in the progression of cancers by influencing the cell cycle and energy metabolism [57]. By monitoring the cell cycle, checkpoints come into effect and contribute to cell cycle arrest when abnormal or incomplete cell cycle events such as DNA damage occur [58, 59].

As ΔN isoforms of p63, ΔNp63, which can be deubiquitinated by USP28, can inhibit apoptosis and cell cycle arrest [15, 60, 61]. In lung squamous cancer cells, USP28 is frequently upregulated, and the level of ΔNp63 is also elevated. Moreover, compared with the control group, cells with depletion of ΔNp63 displayed a reduction in squamous cancer cell proliferation and mild accumulation of cells in S-phase. USP28 depletion had a very similar effect on cell cycle progression and cell proliferation, which could be restored by exogenous ΔNp63 [15]. Furthermore, p53 is a tumor suppressor, and a previous study showed that ΔNp63 can inhibit the transactivation of p53 by binding to the tumor protein p53 (TP53) gene at proapoptotic gene promotors and then inactivating it [15, 60, 62], suggesting that the function of USP28 in squamous cancer cells may be mediated by p53. However, a previous investigation showed that loss of USP28 did not contribute to the activation and alteration of endogenous TP53, suggesting that cell proliferation and cell cycle progression promoted by ΔNp63 are not relevant to p53 [15, 35]. Although the functions of ΔNp63 are independent of p53, these results indicate that USP28 promotes the cell cycle through its impacts on ΔNp63 and then accelerates cell proliferation. In another study, a reduction in G0/G1-phase cells and an increase in S-phase cells were observed in pancreatic cancer cells with ectopic expression of USP28, and this advancement in cell proliferation was mediated by the deubiquitination of Forkhead box M1 (FOXM1), which is induced by USP28 [4].

In high-grade serous ovarian cancer cases, Cyclin E1 is connected with chromosomal instability, the overexpression of which is associated with an increased copy number of USP28, although the effect induced by alteration of USP28 is relatively weaker than CCNE1 (encodes Cyclin E1) amplification [34, 63]. In addition to influencing chromosomal instability, Cyclin E1 possesses the capacity to promote cell cycle progression due to its transition stimulation function between G1/S phases [64]. That is, USP28 may facilitate the cell cycle and cell proliferation by increasing the level of Cyclin E1. In addition, the expression of D-type Cyclins, including Cyclin D1, can sense mitogenic signals and further bind and activate Cyclin-dependent kinase-6 (CDK6) [65]. The activation of the Cyclin D1-CDK6 complex during the G0-to-S transition can be reduced when c-Myc, which is one of the substrates of USP28, is lost [29, 66]. Furthermore, a recent investigation showed a reduction in c-Myc, Cyclin D1 and CDK6 in si-USP28-treated lung cancer cell lines [14]. Hence, USP28 can facilitate the progression of the cell cycle through its indirect stimulation of the Cyclin D1-CDK6 active complex.

Additionally, inhibition of HDAC5 gives rise to abundant cells remaining in the G1 phase and relatively fewer cells in the S phase, which can be reversed by overexpression of the USP28 substrate LSD1 [55]. Therefore, mediated by LSD1, USP28 can accelerate the cell cycle and promote cell proliferation. In addition, Claspin, which was discovered as a protein required for the activation of checkpoint kinase 1 (Chk1), can be deubiquitinated by USP28 through reversing the effect of ubiquitin ligase APC/C^cdh1^ [67, 68]. A previous study showed that, in ovarian clear cell carcinoma cells with DNA damage, knockdown of USP28 reduced both Claspin and Chk1 levels [37]. Thus, deubiquitination of Claspin caused by USP28 contributes to cell cycle arrest, which maintains ovarian clear cell carcinoma cell viability in response to a genotoxic stress [68]. Thus, although USP28 contributes to cell cycle arrest here, it actually functions in tumor promotion and contributes to the drug resistance.

Aerobic glycolysis refers to the consumption of much more glucose in cancer cells than in normal cells, and despite oxygen-rich conditions, glucose is predominantly metabolized by glycolysis, which meets the requirements for cell proliferation [69]. Participating in aerobic glycolysis, FOXC1 belonging to the Forkhead box family can be deubiquitinated by USP28, deregulation of which can bring about the advancement of cancers [70, 71]. A previous study demonstrated that FOXC1 participates in decreasing the expression of fructose-1,6-bisphosphatase 1 (FBP1). In addition, FBP1 is a glycolytic enzyme, and a lack of FBP1 contributes to the appearance of aerobic glycolysis enhancement in colorectal cancer [71]. Accordingly, the inhibited degradation of FOXC1 mediated by USP28 caused the suppression of FBP1, thus contributing to the acceleration of cell proliferation. Other glycolytic regulators, such as hexokinase 2 (HK2), the M2 isoform of pyruvate kinase (PKM2) and LDHA, that can accelerate aerobic glycolysis can be downregulated after the inhibition of USP28 in tumors. Moreover, this process is realized with the mediation of the USP28 substrate c-Myc due to its stimulation of the expression of HK2, PKM2 and LDHA [14, 29, 72, 73].

In addition to promoting the cell cycle and enhancing aerobic glycolysis, USP28 can also stimulate cell proliferation. For instance, loss of USP28 in nude mice resulted in delayed tumor growth of non-small cell lung cancer, which was mediated by the attenuation of STAT3 (a molecule that can be deubiquitinated by USP28) signaling. Similar to the results in nude mice, suppression of cell growth occurred in human non-small cell lung cancer cell lines lacking USP28 as well [33]. In addition, Lin28A, which can be deubiquitinated by USP28, is associated with cell proliferation. Besides, the proliferative behavior mediated by Lin28A is strengthened by USP28 [74].

#### Initiating invasion and metastasis in cancer

As the dominant reason for cancer lethality, metastasis is a process involving the transition of cancer cells from their primary lesion to distant organs. A number of cell mechanisms, such as invading through or colluding with stroma, are related to the transfer of cells [75, 76]. Epithelial–mesenchymal transition (EMT), which refers to a process in which cells lose epithelial characteristics and acquire mesenchymal features, has been demonstrated to play a significant role in the metastatic process of cancer cells [77, 78]. Moreover, N-cadherin is a biomarker of EMT and is significantly correlated with the progression of cancer. Meanwhile, loss of E-cadherin is always related to malignant cells with higher invasiveness and lower levels of differentiation [79, 80]. USP28 has the capacity to stabilize Snail proteins (including Snail, Slug, and Smuc), which can markedly induce EMT by repressing epithelial markers such as E-cadherin [80–82]. In addition, the USP28 substrate LSD1 is indispensable in Snail-mediated transcriptional repression of E-cadherin and other EMT-related molecules [81]. Furthermore, a study showed that loss of USP28 resulted in a reduction in N-cadherin mRNA levels and an increase in E-cadherin mRNA levels in gastric cancer cell lines, indicating that USP28 can positively regulate the process of EMT in gastric cancer [83]. The results above suggest that USP28 may stimulate EMT via its deubiquitination of LSD1 and Snail and then suppress EMT-related molecules.

In addition to the molecules involved in EMT, other molecules, such as Lin28A, which is a crucial regulator of invasiveness, can also accelerate metastasis [84]. In NCCIT cells, wound healing assays and Matrigel cell invasion assays revealed that the USP28 activates the process of metastasis through its deubiquitination of Lin28A [74]. Furthermore, in breast cancer, LSD1 can repress the expression of the tight junction protein claudin-7 (CLDN7) [81]. Loss of CLDN7 facilitates the dissemination of cancer cells [85]. Therefore, the indirect suppression function of USP28 in CLDN7, which is mediated by LSD1, can make a large difference in promoting invasiveness [55].

#### Inhibiting cancer cell differentiation

Cell fate decisions such as cell differentiation, maintenance and self-renewal of stem cells are regulated by Notch signaling. For example, the abrogation of Notch signaling can cause depletion of the stem cell pool of the intestine [86–88]. Notch1 is identified as one of the Notch receptors, whose active form NICD1 is one of the substrates of USP28. NICD1 was observed to be decreased in USP28 knockdown animals, suggesting that the lack of USP28 contributes to the decrease in Notch signaling [35, 89, 90]. Therefore, USP28 can maintain Notch signaling, through which cell differentiation is inhibited.

Moreover, LSD1, which can be deubiquitinated by USP28, has the capacity to regulate pluripotency markers such as octamer-binding transcription factor 4 (Oct4) and SRY-related HMG box-containing 2 (SOX2) and the differentiation gene p21^Cif1/Waf1^ [9, 83, 91]. In breast cancer cell lines with USP28 knockdown or LSD1 knockdown, p21^Cif1/Waf1^ was upregulated, while SOX2 and Oct4 were downregulated. Moreover, the results imply that, mediated by LSD1, USP28 can act as a suppressor of differentiation, and the functions of LSD1 on differentiation genes are direct and indirect for controlling pluripotent genes [9]. In addition to the cell proliferation and metastasis promotion function of Lin28A mentioned before, it suppresses endogenous lethal-7 (let-7) miRNAs as well. Furthermore, the procedure can be enhanced by USP28 [74]. Let-7 is a tumor suppressor that also plays an essential role in the acceleration of cell differentiation [92, 93]; hence, we infer that USP28 may inhibit cell differentiation through its indirect function on let-7.

### Inducing tumor angiogenesis

Angiogenesis refers to the process of new capillaries growing from preexisting blood vessels, which is crucial for growth and metastasis for many tumors, such as pancreatic cancer [94, 95]. Hypoxia inducible factor-1α (HIF-1α) is a crucial component in the process of hypoxia-mediated angiogenesis, and its ubiquitination catalyzed by FBW7 can be turned over by USP28 [25, 96]. In an in vitro Matrigel assay, knockdown of USP28 resulted in decreased capillary-like structure formation under hypoxia. Moreover, the formation of capillary-like structures induced by USP28 was inhibited when HIF-1α was knocked down under normoxia or hypoxia [25]. Moreover, in esophageal cancer, human esophageal cancer cells showed a reduction in HIF-1α in c-Myc knockdown cells but increased HIF-1α when c-Myc was upregulated, suggesting that c-Myc can facilitate the accumulation of HIF-1α [97]. USP28 can induce tumor angiogenesis directly and indirectly through the deubiquitination of HIF-1α and c-Myc.

### Maintaining cancer stem cell-like characteristics

The cancer stem cell (CSC) hypothesis assumes that there exists a subpopulation of neoplastic cells that can asymmetrically divide, among which some remain as CSCs and others differentiate into neoplastic cells [98]. In addition to the function of renewing themselves, CSCs share many other properties with normal stem cells, such as the expression of cell markers such as CD44 [99, 100]. Following the upregulation of USP28 induced by ATG7, CD44 can be deubiquitinated and stabilized by USP28, which mediates the enhancement of the cancer stem cell-like properties of bladder cancer. A study showed that bladder cancer cells treated with a CD44 inhibitor had a tendency to reduce the sizes and numbers of tumorospheres [43], indicating that USP28 can play a vital role in tumor progression by mediating CD44.

Additionally, instead of being required for the activation of EMT, SLUG, a Snail protein, can maintain CSCs, such as exerting effects in lung cancer with the mediation of SOX9 [101]. Furthermore, in addition to being stabilized by USP28 directly, SLUG can also be induced by c-Myc, which is a substrate of USP28, the augmentation of which can induce the maintenance of CSCs [6, 82]. Consequently, USP28 functions on SLUG directly or indirectly to maintain CSC-like characteristics. Furthermore, USP28 and LSD1 were both higher in cells isolated from a tumor model which contributes to the expansion of mammary stem cells. Knockdown of USP28 could induce the differentiation of CSCs while attenuate their self-renewal, which can be reversed by LSD1 expression. Accordingly, the USP28-LSD1 axis can play a vital role in the protection of CSC characteristics [9].

### Tumor-suppressive role of USP28 in carcinoma

**Fig. 5:**
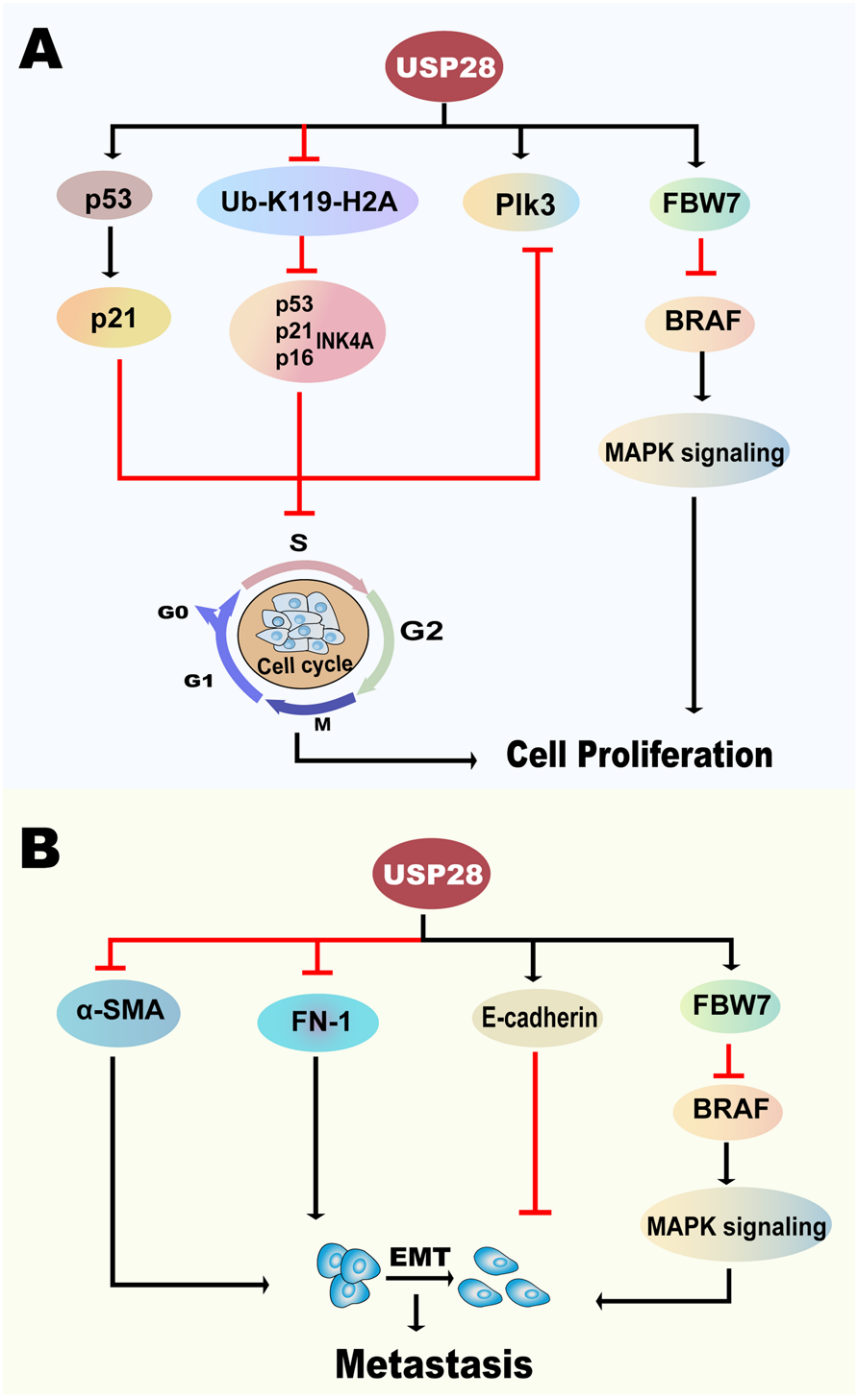
The underlying mechanisms of USP28 in suppressing cancer progression. USP28 can suppress the development of cancer through deubiquitinating several substrates related to tumor inhibition. **A** USP28 deubiquitinates tumor suppressors such as p53 and leads to the cell cycle arrest which can induce inhibition of cancers. **B** USP28 weakens EMT and leads to the suppression of cancer metastasis. *BRAF* v-raf murine sarcoma viral oncogene homolog B, *EMT* epithelial-mesenchymal transition, *FBW7* F-box and WD repeat domain-containing protein 7, *FN-1* fibronectin 1, *MAPK* mitogen-activated protein kinase, *Plk3* polo-like kinase 3, *α-SMA* α-smooth muscle actin, *ub-K119-H2A* ubiquitination at K119 of histone H2A, *USP28* ubiquitin-specific protease 28

The tumor-promoting role of USP28 and relevant investigations are currently dominant; nevertheless, several studies have demonstrated its oncostatic effects. Although USP28 can deubiquitinate several molecules such as p53 to suppress carcinogenesis [26], only melanoma and breast cancer have been demonstrated to have a positive relationship between prognosis and the level of USP28 [10, 16]. According to the investigation results, the oncostatic function of USP28 may occur based on the specific cancer, specific cell lines or specific molecules, which still needs further research and is significant for targeting USP28 for therapy. Then, we will introduce the recent investigation on the suppressive function of USP28 on tumors (Fig. 5; Table 2).

#### Inhibiting cell proliferation in carcinogenesis

The tumor suppressor p53, which participates in cell cycle arrest, cell apoptosis and DNA repair, has been demonstrated to be deubiquitinated and stabilized by USP28 [102, 103]. A study has demonstrated that p53 can not only induce cell cycle arrest but also lead to the maintenance of chromosomal integrity and improve the survival of damaged cells due to its function in the transcriptional activation of p21, which can give rise to G1 arrest and G2/S arrest [104, 105]. Furthermore, by reducing the activation of USP28, caspase-8 has the capacity to decrease the stabilization of p53 and then enhance cell proliferation. Knockdown of caspase-8 can induce the apoptosis of cancer cells with proficient p53 while making no difference to cancer cells without p53 expression [26]. Thus, USP28 functions as a tumor suppressor here. In addition to molecules such as p53 and p21, which are relevant to the cell cycle, p16^INK4A^ is also a tumor suppressor and participates in the regulation of the cell cycle [106]. Elevated ub-K119-H2A has the capacity to reduce p53, p21 and p16^INK4A^, indicating that USP28, which can deubiquitinate and lead to a decrease in ub-K119-H2A, has the ability to enhance the suppression of carcinogenesis by mediating p53, p21 and p16^INK4A^ [32].

Polo-like kinase 3 (Plk3) has been shown to participate in numerous cellular processes, such as DNA damage, mitotic stress and hypoxia stress. In these processes, activated Plk3 has the capacity to phosphorylate many molecules, such as Chk2, p53 and HIF-1α, which contributes to their activation or inactivation and then results in cell cycle arrest and apoptosis. The results indicate that Plk3 plays a crucial role in suppressing tumors, especially in lung cancer [107–110]. Accordingly, USP28 can antagonize the ubiquitination process of Plk3 mediated by SIAH2 [111], which may stimulate cell cycle arrest, promote cell apoptosis and inhibit angiogenesis.

Except for the mechanisms discussed above, v-raf murine sarcoma viral oncogene homolog B (BRAF) mutation is an important gene alteration in cancers of diverse tissues, and it appears in almost 60% of melanomas [112]. In tumors possessing the BRAF V600E mutation, cell proliferation and evasion of apoptosis are promoted by increased extracellular signal-regulated kinase (ERK) signaling. Moreover, ERK signaling can also enhance the mitogen-activated protein kinase (MAPK) cascade, which can promote evasion from G2 arrest [113, 114]. USP28 can antagonize the process of FBW7 autoubiquitination, which can promote the degradation of BRAF and then lead to the attenuation of MAPK signaling [16, 115], suggesting that USP28 can suppress cell proliferation.

#### Suppressing tumor invasion and metastasis

In addition to the decrease in E-cadherin, upregulation of the mesenchymal marker α-smooth muscle actin (α-SMA) is a feature of EMT [116]. A previous study demonstrated that although knockdown of USP28 contributes to the increase in E-cadherin and promotion of gastric cancer invasion [83], accumulation of α-SMA and reduction of E-cadherin appeared in breast cancer cell lines without USP28 [10]. In addition to α-SMA, fibronectin 1, which can also lead to the promotion of EMT, was reduced in breast cancer cell lines overexpressing USP28, while increased levels of E-cadherin were observed [40]. Hence, USP28 plays a significant role in inhibiting EMT in breast cancer. Moreover, the activation of MAPK-ERK can be triggered by Twist, which then gives rise to the enhancement of EMT [117]. Therefore, the attenuation of BRAF and MAPK signaling caused by USP28 can inhibit EMT as well [16].

### Relationship between USP28 and tumor prognosis

In lung squamous cancer, a study containing 300 samples displayed an elevated level of USP28 in cancer tissues in comparison with normal tissues [15]. Similarly, a higher level of USP28 appeared in high-grade glioma samples compared with glioma tissues with lower pathologic grade [12]. Moreover, Kaplan‒Meier survival analysis demonstrated that overexpression of USP28 was associated with shorter overall survival in cancers, including non-small cell lung cancer [13, 15], glioma [12] and hepatocellular carcinoma [118]. Additionally, despite the suppressing function of p53 on carcinogenesis, high expression of transcriptionally inactive p53 contributes to the migration of cancers [119]. Thus, bladder cancer showed a negative relationship between USP28 and prognosis due to the stabilization of transcriptionally inactive p53 caused by USP28 [120, 121].

Contrary to the results described above, a study in a cohort composed of 72 women suffering from breast cancer suggested that a higher level of USP28 was associated with a lower cancer grade. Moreover, cancer-specific survival and disease-free survival analyzed by the Kaplan-Meier method reflected a positive survival trend corresponding to the level of USP28 [10]. Additionally, in melanoma, MANOVA identified USP28 as an independent prognostic factor for survival, and a study showed that low expression of USP28 was correlated with poor overall survival [16]. Thus, during past investigations concerning the relationship between USP28 and tumor prognosis, the negative role of USP28 in prognosis has been discussed dominantly, while the positive role has also attracted scientists’ attention.

### Roles of USP28 in influencing therapy resistance

Recently, epigenetic therapy with 5’-azacytidine (5’AZA) has been demonstrated to prolong the overall survival of patients suffering acute myeloid leukemia [122]. Nevertheless, silencing of the UCK family can give rise to a blunted response to 5’AZA due to its significance in the activation and metabolism of 5’-AZA in vitro [123]. Thus, through restraining the ubiquitination and degradation of UCK1, overexpression of USP28 resulted in increased effects of 5’AZA on the apoptotic process in acute myeloid leukemia cells. In addition, the results in the intravenous acute myeloid leukemia mouse model showed that the growth of acute myeloid leukemia cells with upregulation of USP28 was suppressed after treatment with 5’-AZA, especially compared with cells without USP28 [27]. Similarly, sensitivity to vemurafenib-induced tumor shrinkage can be reduced by depletion of USP28 in immunodeficient mice injected with melanoma cells [16].

Except for promoting the sensitivity of the drug to cancer, opposite influences of USP28 on cancer therapy are currently being revealed. For example, experiments conducted in human esophageal cancer cells proved that a higher level of USP28 in cells treated with radiotherapy leading to increasing survival of cancer cells [97], suggesting that USP28 contributes to radiotherapy resistance in esophageal cancer. Moreover, the USP28 substrate STAT3 participates in the drug resistance of cancer therapy, suggesting that USP28 may play an essential role in therapy resistance in non-small cell lung cancer [33, 124] (Table 2).

### Potential drugs related to USP28 for cancer therapy

Following the introduction of the functions of USP28 in cancer prognosis and therapy resistance, potential future drugs that inhibit cancer progression by targeting USP28 are discussed. Streptoglutarimide H (SGH) is isolated from a culture of *Streptomyces* sp. ZZ741 (marine-derived actinomycete) in rice medium [125], lung cancer cells treated with which displayed inhibited cell proliferation with IC_50_ values of 1.69–5.24 µM. Moreover, a remarkable decrease in USP28 and its substrates, such as c-Myc and Cyclin D1, was shown in lung cancer cells treated with SGH, suggesting that the anticancer function of SGH may be mediated by the downregulation of USP28 [14].

In addition to SGH, AZ1 is another inhibitor of USP28 that is quite significant for the treatment of lung cancer. Cultured in the presence of AZ1, A-431 cells displayed a reduction in the activation and abundance of USP28. In addition, in wild-type C57BL/6J mice transplanted with murine lung tumor cells, compared with the control mice, AZ1 reduced the tumor burden in a dose-dependent manner and blocked the activation and reduced the abundance of USP28 [15].

Additionally, a new series of [1, 2, 3] triazolo [4,5-d] pyrimidine derivatives have been identified as inhibitors of USP28, among which compound 19 bearing the 4-chlorobenzyl group is the best, reaching the expectation of targeting USP28. A study showed that compound 19 had a remarkable suppressive effect on cancer cells with USP28 overexpression. Furthermore, compound 19 suppressed colony formation, cell proliferation, the cell cycle at S phase, migration, and the EMT process in vitro, and its cytotoxic effect was partially dependent on the inhibition of USP28 [126].

Moreover, a previous study showed that lanatoside C could induce apoptosis and then inhibit cell proliferation by promoting c-Myc degradation, which was mediated by the ubiquitin‒proteasome pathway. Although the ubiquitination of c-Myc induced by lanatoside C can be turned over by USP28, what was further demonstrated is that lanatoside C can attenuate the interaction between USP28 and c-Myc. Therefore, lanatoside C promoted the advancement of cancer indirectly via its influence on USP28 function [127]. Similar to lanatoside C, caffeic acid 3,4-dihydroxyphenethyl ester (CADPE), which is derived from *Teucrium pilosum,* can downregulate USP28 and then contribute to the decrease in c-Myc. Moreover, CADPE not only showed effective anti-leukemia activity in some leukemia cell lines but also displayed a higher safety level for normal cells compared with the positive groups treated with imatinib and arsenic trioxide due to its suppression of c-Myc [30, 128]. Thus, CADPE can also function as an inhibitor of cancer because of its antagonization of deubiquitination caused by USP28. Additionally, iorhapontigenin (ISO) is isolated from the Chinese herb *Gnetum cleistostachyum*, and a reduction in the activation of the USP28 mRNA 3’-UTR appeared in bladder cancer cells treated with ISO, through which the suppression of bladder cancer was promoted [36, 129]. Moreover, vismodegib and FT206 can target USP28 and attenuate its activation, leading to the inhibition of colorectal cancer and lung squamous cell carcinoma, respectively [21, 130] (Table 3).

**Table 3:** Drugs related with USP28 for therapy

USP28 inhibitor	Cancer type	Cell lines	Kd value	IC_50_ value	References
[1,2,3]triazolo[4,5- d]pyrimidine (Compound 19)	Gastric cancer	HGC-27 cells	40 nmol/L	1.10 ± 0.02 μmol/L	[126]
Streptoglutarimide H	Lung cancer	PC9 and H157 cells	–	–	[14]
AZ1	Lung squamous cancer	A-431 cells	–	18.8 μM	[15]
Lanatoside C	Gastric cancer	HEK 293 cells	–	–	[127]
Isorhapontigenin	Bladder cancer	T24T cells	–	–	[36]
Caffeic acid 3,4-dihydroxyphenethyl ester	Leukemia	Jurkat cells	–	–	[30]
Vismodegib	Colorectal cancer	Ls174T and HCT116 cells	1.42 ± 0.18 μM	4.41 ± 1.08 μM	[21]
FT206	Lung squamous cancer	H520 cell	–	EC_50_ ~ 1–3 μM	[130]

*SGH* Streptoglutarimide H, *ISO* Isorhapontigenin, *CADPE* Caffeic acid 3,4-dihydroxyphenethyl ester

## Discussion

As a deubiquitinase, USP28 can participate in the deubiquitination of abundant molecules related to cancers, including LSD1 [55] and p53 [26]. Moreover, in the process of exploring the functions of USP28, investigations have reported that USP28 has the capacity to influence significant processes such as the DNA damage response and cell proliferation [7, 32]. Based on these studies, the relationship between USP28 and carcinogenesis is gradually exposed, which is that USP28 can not only accelerate but also suppress the progression of cancers [10]. Therefore, whether USP28 can be a therapeutic target or whether USP28 inhibitors such as vismodegib can be used in cancer treatment has attracted the attention of scientists [21].

With the advancement of the investigation of the relationship between USP28 and cancers, numerous studies have illustrated that USP28 can facilitate carcinogenesis by playing a role in cancer hallmarks involving cell proliferation, cell metastasis, cell differentiation, angiogenesis and CSC-like characteristics [15, 25, 35, 36, 74]. In addition to its significance in oncogenic functions attracting the attention of researchers, the anti-carcinoma effects of USP28 have also attracted attention. However, compared with the abundant investigations concerning its functions in promoting cancer progression, only a few studies have reported the oncostatic functions of USP28, such as its suppression of cell proliferation and invasion [10, 68]. Therefore, further investigation should be conducted to explore its anticancer functions and reveal a more accurate relationship between USP28 and cancers.

As mentioned before, USP28 can deubiquitinate diverse substrates and then display two opposite roles in one kind of cancer, such as breast cancer. However, as the search showed, the higher level of USP28 still has nonnegligible value in predicting better survival and TNM classification of breast cancer [9, 10]. That is, the role of USP28 in breast cancer is to suppress cancers. Nevertheless, why its inhibitory function is ascendant in breast cancer remains unknown. Whether the diverse functions displayed in different cancers are related to specific genetic backgrounds, tissue specific, caused by diverse levels of related molecules such as FBW7, or relevant to diverse pathologic classifications of cancers can be a future direction, which may be instructive for targeting USP28 in the treatment of cancers.

Ubiquitination plays a significant role in the process of protein interactions, localization and enzymatic activities, thus impacting cellular processes covering transcription, DNA damage response, cell cycle and endocytosis [131]. Serving as antagonists of ubiquitination, deubiquitinases maintain the balance of these processes related to ubiquitination, contributing to the stability of the whole body, similar to the functions of dephosphorylation [2, 132]. Additionally, although some drugs, such as AZ1 [15], have been demonstrated to suppress the advancement of cancers under the mediation of USP28, clinical trials and investigations concerning targeting USP28 for cancer therapy are still insufficient. Hence, although inhibitors of USP28 display noteworthy results in inhibiting cancer, possible side effects of the drugs used clinically in the future are inevitable. This highlights the importance of exploring deeper mechanisms to clarify whether inhibitors of USP28 are safe for patients suffering from cancers.

A study showed that beyond the conserved UBP domains, USP28 shared amino acid identities accounting for 51% with USP25 [3]. Whether USP28 can deubiquitinate the substrates of USP25, such as the BCR-ABL protein, needs further exploration [133]. Moreover, among the drugs possessing the ability to target USP28 and then contribute to the inhibition of cancers, both AZ1 and vismodegib target not only USP28 but also USP25 [21, 134]. As another deubiquitinase, USP25 can take part in many diseases, not only cancers but also Alzheimer’s disease and antiviral immunity [135–137], suggesting that inhibitors of both USP25 and USP28 may influence other biological or pathological processes mediated by the lack of USP25. Thus, more drugs with better selectivity must be explored deeply due to their unpredictable impacts on normal biological functions.

Combination of checkpoint inhibitors and oncogene-targeted drugs in the treatment of cancers might improve the management of cancers [138]. For example, renal cell carcinoma patients treated with sunitinib exhibited increased intratumoral T cells, increased PD-L1 expression in the tumor, and increased PD-1 expression on tumor-infiltrating lymphocytes [139]. Thus, whether inhibitors of USP28 can also be used in combination with immunotherapy is a valuable direction for clinical application. Moreover, a study has shown that in patients who can benefit from combination therapy, BRAF mutation is a significant gene mutation [138]. Furthermore, it has been suggested that inhibition of MAPK signaling may contribute to the potentiation of immunotherapy effects [140]. In addition, USP28 function results in the attenuation of MAPK signaling [16, 115]. Hence, more investigations are needed to expose the impact of the combination of USP28 inhibitors and immunotherapy.

## Conclusions

In conclusion, USP28, a deubiquitinase found due to its homology with USP25, mainly regulates the ubiquitination degradation of many proteins. The results generated after its regulation on the proteins include promoting cell proliferation, initiating invasion and metastasis, stimulating cell survival, inhibiting cell differentiation, inducing angiogenesis and maintaining CSC-like features, all of which contribute to the progression of tumors. Meanwhile, its deubiquitinating functions on some anticancer proteins reveal its nonnegligible roles in cell proliferation inhibition and suppression of invasion and metastasis of carcinomas. The expression of USP28 is precisely controlled by its upstream molecules, including microRNAs such as miR-500a-5p and other molecules such as KRAS. In addition, other posttranscriptional modifications, such as SUMOylation, are also involved in regulating the stabilization of USP28. Moreover, the pro- and anticancer impacts of USP28 signify its potential in clinical practice, especially as a therapeutic target in the future. Its crucial roles in influencing the prognosis and sensitivity of cancers toward diverse therapies have also been demonstrated. However, a number of relevant mechanisms remain unclear, and the effects and safety of its clinical application remain in a situation requiring many more experiments.

## Data Availability

Not applicable.

## Abbreviations

ATG: Autophagy related-protein
AUF1: ARE/poly(U)-binding/degradation factor 1
5’AZA: 5’-Azacytidine
BRAF: V-raf murine sarcoma viral oncogene homolog B
CADPE: Caffeic acid 3,4-dihydroxyphenethyl ester
CD44: Cluster of differentiation-44
CDK6: Cyclin-dependent kinase-6
Chk1: Checkpoint kinase 1
CLDN7: Tight junction protein claudin-7
CSC: Cancer stem cell
DUB: Deubiquitinating enzyme
EMT: Epithelial–mesenchymal transition
ERK: Extracellular signal-regulated kinase
FBP1: Fructose-1,6-bisphosphatase 1
FBW7: F-box and WD repeat domain-containing protein 7
FOX: Forkhead box
GSK-3β: Glycogen synthase kinase-3β
HDAC: Histone deacetylases
HIF-1α: Hypoxia-inducible factor-1α
HK2: Hexokinase 2
ISO: Isorhapontigenin
KLHL2: Kelch-like protein 2
LDHA: Lactate dehydrogenase A
let-7: Lethal-7
LSD1: Lysine specific demethylase 1
MAPK: Mitogen-activated protein kinase
MDM2: Mouse double minute 2 protein
NICD1: NOTCH1 intracellular domain
Oct4: Octamer-binding transcription factor 4
PKM2: M2 isoform of pyruvate kinase
Plk3: Polo-like kinase 3
PTM: Post-translational modification
SENP: SUMO-specific protease
SGH: Streptoglutarimide H
SIM: SUMO-interaction motif
α-SMA: α-Smooth muscle actin
SOX: SRY-related HMG box-containing
STAT3: Signal transducer and activator of transcription 3
SUMO: Small ubiquitin-like modifier
Tet1: Ten-eleven translocation1
TP53: Tumor protein p53
UBA: Ubiquitin-associated domain
ub-K119-H2A: Ubiquitination at K119 of histone H2A
UCID: USP28 catalytic domain inserted domain
UCK: Uridine-cytidine kinase
UIM: Ubiquitin-interaction motif
USP: Ubiquitin-specific protease
